# Anti-Correlation between the Dynamics of the Active Site Loop and C-Terminal Tail in Relation to the Homodimer Asymmetry of the Mouse Erythroid 5-Aminolevulinate Synthase

**DOI:** 10.3390/ijms19071899

**Published:** 2018-06-28

**Authors:** Insung Na, Dominique Catena, Min J. Kong, Gloria C. Ferreira, Vladimir N. Uversky

**Affiliations:** 1 Department of Molecular Medicine, Morsani College of Medicine, University of South Florida, Tampa, FL 33612, USA; Insung.Na@childrens.harvard.edu (I.N.); dcatena@health.usf.edu (D.C.); mkong@health.usf.edu (M.J.K.); gferreir@health.usf.edu (G.C.F.); 2 Department of Chemistry, College of Arts and Sciences, University of South Florida, Tampa, FL 33612, USA; 3 USF Health Byrd Alzheimer’s Research Institute, Morsani College of Medicine, University of South Florida, Tampa, FL 33612, USA; 4 Institute for Biological Instrumentation of the Russian Academy of Sciences, Pushchino, Moscow 142290, Russia

**Keywords:** ALAS, homodimer asymmetry, molecular dynamics, intrinsically disordered region, anti-correlated dynamics

## Abstract

Biosynthesis of heme represents a complex process that involves multiple stages controlled by different enzymes. The first of these proteins is a pyridoxal 5′-phosphate (PLP)-dependent homodimeric enzyme, 5-aminolevulinate synthase (ALAS), that catalyzes the rate-limiting step in heme biosynthesis, the condensation of glycine with succinyl-CoA. Genetic mutations in human erythroid-specific ALAS (ALAS2) are associated with two inherited blood disorders, X-linked sideroblastic anemia (XLSA) and X-linked protoporphyria (XLPP). XLSA is caused by diminished ALAS2 activity leading to decreased ALA and heme syntheses and ultimately ineffective erythropoiesis, whereas XLPP results from “gain-of-function” ALAS2 mutations and consequent overproduction of protoporphyrin IX and increase in Zn^2+^-protoporphyrin levels. All XLPP-linked mutations affect the intrinsically disordered C-terminal tail of ALAS2. Our earlier molecular dynamics (MD) simulation-based analysis showed that the activity of ALAS2 could be regulated by the conformational flexibility of the active site loop whose structural features and dynamics could be changed due to mutations. We also revealed that the dynamic behavior of the two protomers of the ALAS2 dimer differed. However, how the structural dynamics of ALAS2 active site loop and C-terminal tail dynamics are related to each other and contribute to the homodimer asymmetry remained unanswered questions. In this study, we used bioinformatics and computational biology tools to evaluate the role(s) of the C-terminal tail dynamics in the structure and conformational dynamics of the murine ALAS2 homodimer active site loop. To assess the structural correlation between these two regions, we analyzed their structural displacements and determined their degree of correlation. Here, we report that the dynamics of ALAS2 active site loop is anti-correlated with the dynamics of the C-terminal tail and that this anti-correlation can represent a molecular basis for the functional and dynamic asymmetry of the ALAS2 homodimer.

## 1. Introduction

Heme synthesis in metazoan, fungi and α-proteobacteria is a complex, multi-step enzymatic process with 5-aminolevulinate synthase (ALAS) catalyzing the first and rate-limiting step. ALAS uses succinyl-CoA and glycine as substrates to produce 5-aminolevulinate (ALA) [1,2,3,4]. This ALA synthetic route is known as the C4 or Shemin pathway, contrasting with the synthesis of ALA from glutamate, via the C5 pathway, in plants and bacteria [5,6]. Two genes with distinct chromosomic locations encode the two mammalian ALAS isoforms: the housekeeping (ALAS1) and the erythroid-specific (ALAS2) enzymes [1]. Mutations in human ALAS2 yielding diminished ALAS activity or “loss-of-function” are associated with X-linked sideroblastic anemia (XLSA; MIM 300751) [7,8], while “gain-of-function” ALAS2 mutations are present in X-linked protoporphyria (XLPP; MIM 300752) [8,9,10,11,12]. The main clinical feature of XLSA relates to the ineffective erythropoiesis due to decreased ALA and heme syntheses. This results in incomplete maturation of erythrocytes and premature death of the cells within the bone marrow and subsequent iron overload within organs, such as the liver [7,8]. The main clinical hallmark of XLPP, on the other hand, is protoporphyrin IX production far exceeding the amount of ferrous iron (Fe^2+^) and the needs for heme synthesis in the bone marrow [10]. Given this unbalanced ratio of protoporphyrin to Fe^2+^, the heme-synthesizing enzyme, protoporphyrin ferrochelatase, incorporates the alternative metal ion substrate Zn^2+^ into protoporphyrin [13]. Thus, elevated plasma protoporphyrin and Zn^2+^-protoporphyrin levels are observed in XLPP patients [10,13]. All of the identified human ALAS2 mutations associated with XLPP reside in exon 11 encoding the C-terminus of the protein [9,11,14,15]. Nonsense (e.g., c.1642C > T(p.Q548X)) and frameshift (e.g., c.1699-1700delAT(p.M567EfsX2), c.1706-1709delAGTG (p.E569GfsX24), and c.1651-1677del26bp (pS551PfsX6)) mutations yield forms of the ALAS2 protein with either truncated or extended C-termini [11,15,16]. A C-terminal “gain-of-function” domain was narrowed down to a minimal size of 33 amino acids, from Gly544 to Gly576 [15].

Protein conformational transitions play a particularly important role in ALAS function [17,18,19]. Succinyl-CoA binding to the ALAS-glycine complex induces a change from an open to a closed conformation significantly increasing (˃2 × 10^5^-fold) the rate of proton abstraction from glycine and producing a carbanion or quinonoid intermediate [20]. Condensation of succinyl-CoA with the generated quinonoid intermediate prompts a series of chemical steps that culminate with the formation of ALA [17,18]. Returning from the closed to the open and resting state of the enzyme allows release of the ALA product from the ALAS active site and dictates the rate-limiting step of the ALAS catalytic pathway [18,19]. Of relevance, the major protein conformation change is centered on a mobile active site loop [3], located approximately 20 N-terminal amino acids from the “gain-of-function” domain. Saturation mutagenesis of the murine ALAS2 active site loop permitted us to identify ALAS variants that were not only significantly more active than the wild-type enzyme, but also had reactions that were not limited by product release [21]. We proposed that this enhanced activity of the ALAS2 variants was due to an increase in the conformational mobility of the active site loop, which facilitated product release by stabilizing the open conformation of the enzyme [2,21]. Further, XLPP mutations appear to destabilize the succinyl-CoA-induced human ALAS2 closed conformation and accelerate ALA release [9]. The C-terminal region was equally predicted to serve as a site for allosteric interactions, such that ALAS activity can be modulated, and the modulation can go awry or be lost with the XLPP mutations [2,9].

While the importance of the C-terminus in the regulation of the mammalian ALAS function cannot be dismissed, its tertiary structure remains undefined. Structural information on ALAS is limited to the solution structure of the 49 amino acid-long pre-sequence of murine ALAS2 determined by nuclear magnetic resonance (NMR) spectroscopy [22] and the crystallographic structure of *Rhodobacter capsulatus* ALAS [3]. However, the bacterial enzyme lacks the C-terminal tail found in mammalian ALAS2, where the human XLPP mutations appear to cluster [9,14,15,16]. Given this state of affairs, we used bioinformatics and computational biology analyses as the experimental approach to delineate and evaluate the role(s) of the flexibility of C-terminal tail in ALAS structure and dynamics.

The remarkable commonness of intrinsically disordered proteins (IDPs) and proteins containing functionally important intrinsically disordered protein regions (IDPRs) in all proteomes is widely recognized [23,24,25,26,27,28,29,30], although catalytic domains of enzymes represent an exception to the rule of protein intrinsic disorder due to their need of unique structure for catalysis [31,32,33,34,35,36,37,38,39,40,41,42]. Nevertheless, a recent analysis clearly showed that many enzymes contain biologically important IDPRs, with eukaryotic enzymes having a greater intrinsic disorder content than prokaryotic enzymes [43]. Since IDPs/IDPRs are typically characterized by high evolutionary rates that define low conservation of their sequences [44,45,46], it is reasonable to hypothesize that the non-conserved C-terminal tail of human ALAS2 is intrinsically disordered.

Introduction of seven specific mutations in the ALAS2 active site loop yielded an enzyme variant (a “hepta-variant”) with heightened activity and substrate specificity for the two substrates [21]. Using molecular dynamics (MD) simulation analysis, we showed that the introduced mutations affected the dynamic and structural properties of the active site loop; namely, the mutations caused the loop to form a β-strand more frequently than the wild-type loop [47]. Still, the emergence of a β-strand was apparent in the wild-type ALAS2 loop. When these changes in secondary structure occurred in the active site loop of one given chain of the ALAS2 dimer, then the C-terminal tail of the same ALAS2 chain was more dynamic, as judged from visual inspection [47]. Also, the dynamic behavior of the two protomers of the ALAS2 dimer was different, with one being noticeably more mobile than the other [47]. Based on these previous observations, we decided to analyze whether modifications of the ALAS C-terminal tail impacted the dynamics of the hepta-variant active site loop. We hypothesized that loss or extension of the non-conserved human ALAS2 C-terminal tail triggers structural changes in the active site loop, and the dynamics of the ALAS2 active site loop and C-terminal tail are anti-correlated in relation to the homodimer asymmetry. With this study, we addressed our general hypotheses, employing MD simulation analyses to investigate the dynamic behavior of the ALAS2 homodimer. Based on the analysis of the dynamic behavior of HemA monomer (HemAm), ALAS2 monomer without the wild-type C-terminal tail (ALASmWoT), mature wild-type ALAS2 monomer (ALASm), and the wild-type ALAS2 homodimer containing two polypeptide chains (ALASdChA, ALASdChB), we report here that the dynamics of the ALAS2 active site loop is anti-correlated with the dynamics of the C-terminal tail.

## 2. Results

### 2.1. Potential for Formation of a β-Strand in the ALAS2 C-Tail as Revealed Using Bioinformatics

**Figure 1:**
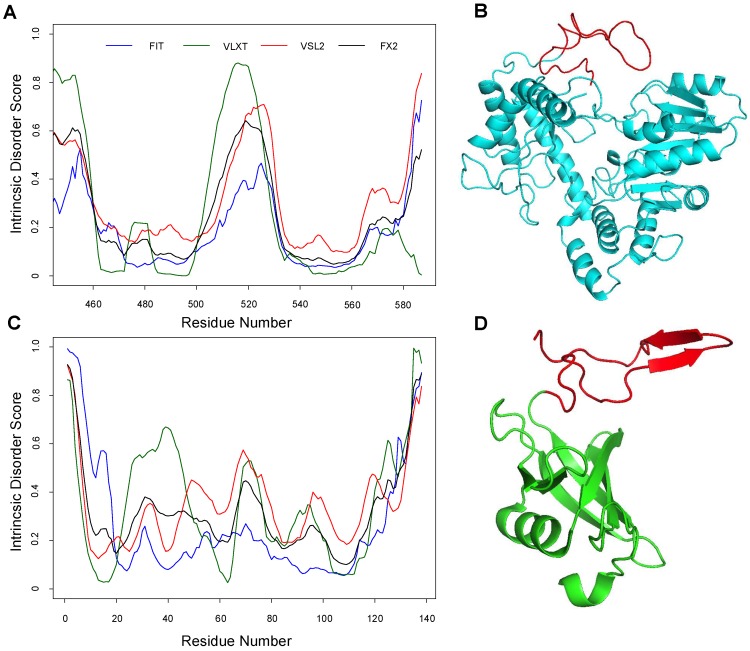
Analysis of a protein homologous to ALAS2 but with a structured C-terminal tail region. (**A**) Evaluation of the intrinsic disorder propensity of ALAS2 by a series of disorder predictors. (**B**) Modelled 3D structure of one of the ALAS2 protomers (ALASdChB) upon MD simulation at 310 K (ALASdChB-310 K 1st cluster). (**C**) Evaluation of the intrinsic disorder propensity of *Methanothermobacter thermautotrophicus* phosphoribosyl-AMP cyclohydrolase HisI by a series of disorder predictors. (**D**) 3D structure of HisI (PDB ID 1ZPS). Intrinsic disorder predispositions of both proteins were evaluated using PONDR^®^ VLXT, PONDR^®^ VSL2, and PONDR^®^ FIT, and the outputs of individual predictors were averaged (FX2). The ALAS2 active site loop and the C-tail of His I are indicated in red in plots (**B**,**D**), respectively.

Before proceeding with the MD simulation to investigate the dynamic behavior of the ALAS2 active site loop and the C-terminal tail, we searched for the potential foldability of the ALAS2 C-terminal tail by analyzing homologous C-terminal regions in other proteins. Amino acid sequence identity between *R. capsulatus* ALAS or HemA (HemA, UniProt ID: P18079) and the mature form of *Mus musculus* ALAS2 (ALAS2, UniProt ID: P08680, residues D79-A587) is 39% [47]. Although the sequence identity between the conserved regions of these two proteins is 48%, murine ALAS2 contains a 35 residue-long C-terminal tail (residues A553-A587), which is missing in HemA. The sequence of the murine ALAS2 C-terminal tail was used in the BLAST search against the UniProt 2017_02 bacterial proteome (UniProtKB Bacteria). Five sequences showed 46% identity with the C-terminal tail of murine ALAS2. Of these, the amino acid sequence of the *Megasphaera elsdenii DSM 20460* histidine biosynthesis bifunctional protein HisIE (UniProt ID: G0VL69) was used for the BLAST search against the UniProt 2017_02 eukaryote proteome (UniProtKB Eukaryota). This search yielded two proteins, *Arabidopsis thaliana* histidine biosynthesis bifunctional protein HisIE (UniProt ID: O82768) and *Kluyveromyces lactis* histidine biosynthesis trifunctional protein (UniProt ID O13417), characterized by 35% and 32% sequence identity to HisIE, respectively. Homology models for these two eukaryotic proteins were previously generated using the X-ray crystal structure coordinates (PDB ID: 1ZPS) of the *Methanobacterium thermoautotrophicum* phosphoribosyl-AMP cyclohydrolase HisI (UniProt ID O26347) [48]. Therefore, our bioinformatics search revealed that *M. thermoautotrophicum* phosphoribosyl-AMP cyclohydrolase HisI, with known X-ray crystal structure, appeared to serve as a suitable candidate for our subsequent studies. In fact, the intrinsic disorder propensity analysis showed that the C-terminal regions of ALAS2 and HisI are characterized by similar disorder profiles, with an intrinsic disorder propensity increase in their last 40 residues (Figure 1). Although the murine ALAS2 C-terminal tail did not form a noticeable ordered secondary structure during the MD simulations, a folded structure with two anti-parallel β-strands was identified in the *M. thermoautotrophicum* HisI C-terminal region (Figure 1).

### 2.2. Higher Backbone RMSD in the ALAS2 C-Tail Than in Other Protein Regions

To evaluate the dynamic properties of four systems: HemA monomer (HemAm), ALAS2 monomer without the wild-type C-terminal tail (ALASmWoT), mature wild-type ALAS2 monomer (ALASm), and the wild-type ALAS2 homodimer containing two polypeptide chains (namely, ALASdChA and ALASdChB for analysis after MD simulation) in different conditions, we performed MD simulations at three different end temperatures (288 K, 300 K, and 310 K) and an explicit solvent. The experimental conditions were as described in [49], and the 288 K temperature was selected because we used this temperature in our previous experimental and computational studies [9,47]. The following analyses of each 100 ns production run were conducted: (i) the atomic RMSD calculation of the backbone atoms (the backbone RMSD), (ii) the residue level RMSF calculation of the backbone atoms (the backbone RMSF), (iii) calculation of the number of active site loop inner hydrogen bonds, (iv) evaluation of the number of formed β-strands, and (v) evaluation of the correlation among the C_α_ atoms focusing on active site loop and C-terminal tail.

The two-dimensional backbone RMSD representation of the results of the MD simulations shows structural deviations from a previously published reference structure [49]. This approach is frequently used for evaluation of the dynamic dissimilarities between the different regions of structure. Specifically, two-dimensional profiles are created from the simulations using the backbone RMSD of two segments as the reaction coordinates. We compared the backbone RMSD of three regions (N-terminal helix, conserved region, and C-terminal tail) of ALASm, ALASdChA, ALASdChB at three different temperatures (288 K, 300 K, and 310 K) (Supplementary, Figure S1). The energy minimized structure of each system served as reference in these experiments.

**Figure 2:**
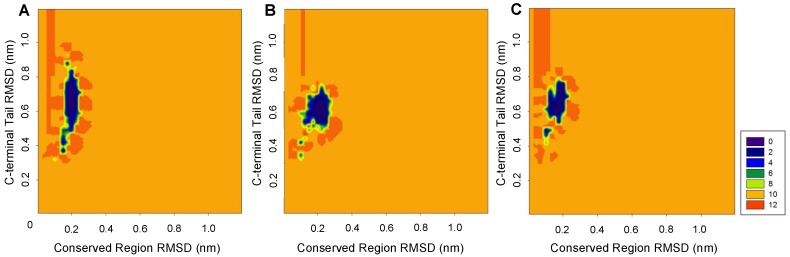
Two-dimensional backbone RMSD representation of the MD simulation results for comparison of the dynamics of the conserved region and the C-terminal tail region of three different ALAS2 systems at 300 K. (**A**) ALASm, (**B**) ALASdChA, (**C**) ALASdChB. The 288 K and 310 K RMSD landscapes also showed similar patterns (Supplementary, Figure S1).

Figure 2 compares distributions of the backbone RMSD values for the conserved region and the C-terminal tail at 300 K. The backbone RMSD of the conserved region was distributed within a relatively narrow interval below 0.3 nm, whereas the distribution of the backbone RMSD of the C-terminal tail was much broader and exceeded 0.3 nm. This indicates that C-terminal tail is highly dynamic and is characterized by a broad RMSD distribution. Among the analyzed systems, ALASm showed a wider C-terminal tail RMSD distribution than ALASdChA or ALASdChB. However, the dynamic behavior of the conserved region was the opposite, since the backbone RMSD distribution for this region in ALASm was narrower than the corresponding distributions recorded for the two other systems. Therefore, we focused on the analysis of structural fluctuations and folding properties of the conserved region and the C-terminal tail of ALAS2, to complement the information on the dynamic behavior of the active site loop next to the strand β13 [3,47].

### 2.3. Fewer Structural Fluctuations in the Conserved Region Than in the C-Terminal Tail of ALAS2 as Deduced from the Backbone RMSF Analysis

**Figure 3:**
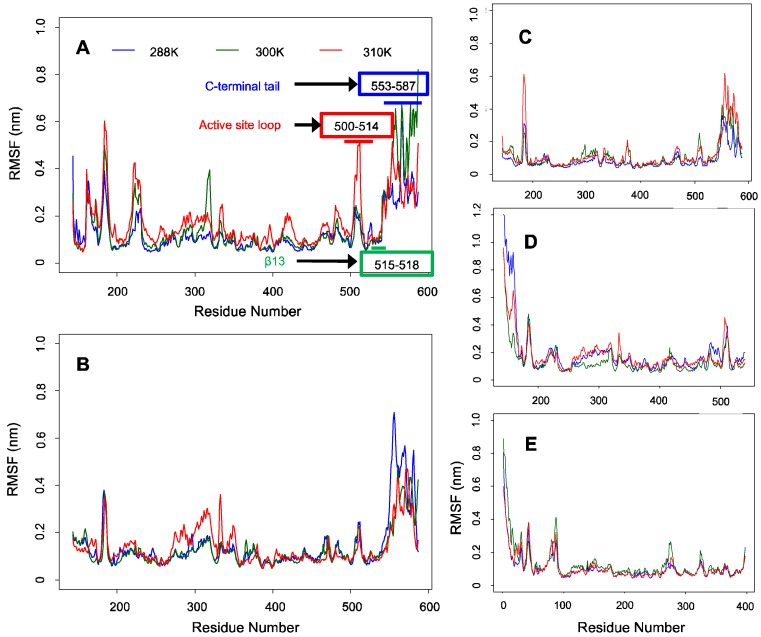
Backbone RMSF plots for ALAS2 and HemAm at three different temperatures. (**A**) ALASm, (**B**) ALASdChA, (**C**) ALASdChB, (**D**) ALASmWoT, (**E**) HemAm.

Backbone RMSF analysis revealed greater fluctuations in the C-terminal tail than in the conserved regions of ALASm, ALASdChA, and ALASdChB (see Figure 3). Although it was not obvious in other cases, the backbone RMSF plots from MD simulations of mALAS at 300 K and 310 K showed a pattern consistent with our hypothesis, given the noticeable anti-correlation between the active site loop and C-terminal tail dynamics. In fact, the level of fluctuation of the active site loop was higher at 310 K than 300 K, but the degree of fluctuation of the active site loop was lower than that of the C-terminal tail at 300 K.

In the case of systems with no C-terminal tails, the N-terminal helix fluctuated more than other regions of the ALASmWoT and HemAm systems. One should note though that other MD simulations did not show this pattern. Based on these observations, it is expected that the dynamics of the N-terminal helix is also anti-correlated with the dynamics of other regions of these proteins. This observation indicates that a dedicated analysis should be conducted to examine whether the anti-correlation in the dynamics between the intrinsically disordered region containing the N-terminal helix and the conserved region of ALAS2 is similar to the dynamic anti-correlation between the C-terminal tail and the conserved region.

### 2.4. Confirmation of the Correlation between the Number of Hydrogen Bonds and the β-Strand-Forming Residues in the ALAS2 Active Site Loop as Revealed by MD Simulations

**Figure 4:**
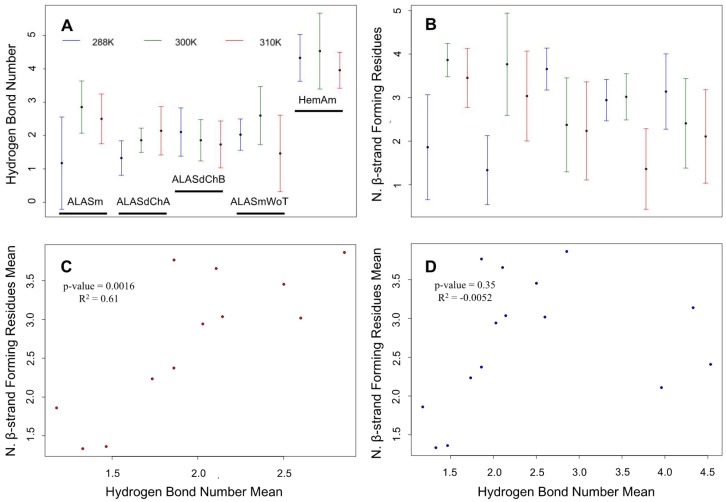
Analysis of the secondary structure propensity of the active site loop in MD simulations of different ALAS2 and HemAm systems. (**A**) The number of hydrogen bonds in the active site loop at different temperatures. (**B**) The number of β-strand forming amino acids in the active site loop at different temperatures. Legends for proteins and color code in plot B are the same as in A. Central dots indicate the mean values, and error bars represent the standard deviations. (**C**) Correlation between the mean hydrogen bond number and the mean number of β-strand forming amino acids for four ALAS2 systems (i.e., ALASm, ALASdChA, ALASdChB and ALASmWot). (*y* = −0.04 + 1.41 *x*, where *y* is the N. β-strand-forming residues mean, and *x* is the mean number of hydrogen bonds) (**D**) Correlation between the mean number of hydrogen bond and the mean number of the β-strand forming amino acid for four ALAS2 systems (i.e., ALASm, ALASdChA, ALASdChB and ALASmWoT). (*y* = 2.22 + 0.20 *x*, where *y* is the mean number of β-strand-forming residues, and *x* is the hydrogen bond number mean. R^2^ is the adjusted r-squared value from R).

The backbone RMSF analysis revealed that among all of the tail-containing systems included in the MD simulation analyses, ALASm at 300 K fluctuated the most in the C-terminal tail region. According to our loop-tail anti-correlated dynamics hypothesis, the ALASm active site loop would have a greater folding propensity at 300 K. Hence, in order to assess whether the dynamics of the ALASm active site loop is anti-correlated with the dynamics of the C-terminal tail, we measured the total number of hydrogen bonds and the number of β-strand-forming residues in the active site loop of the different ALAS systems (i.e., ALASm, ALASdChA, ALASdChB, ALASmWot and HemAm) at different temperatures (i.e., 288 K, 300 K and 310 K). Both the mean numbers of hydrogen bonds and β-strand-forming residues were the highest for ALASm at 300 K (Figure 4A,B). In addition, the visual inspection of representative structures from the top 3 clusters revealed the presence of the β-strand in the active site loop (Supplementary, Figure S2). In fact, ALAS2 MD simulations showed the formation of 1–3 hydrogen bonds between the active site loop and the β13 strand and the presence of 1–4 β-strand-forming amino acids in the active site loop. The MD simulations of the five ALAS systems revealed that HemAm possessed the greatest number of hydrogen bonds (~4) between the active site loop and the β13. In contrast, HemAm had the lowest number of β-strand forming residues (2–3). This structural dynamic behavior was clearly distinct from those for the ALAS2 systems, as inferred from the MD simulations.

Because hydrogen-bonding is a characteristic feature of β-structure formation, we analyzed whether a correlation could be established between the mean values of the number of hydrogen bonds and the mean values of the number of the β-strand-forming amino acids for the different ALAS2 and HemAm systems. Our analysis revealed a significant correlation between these mean values in the case of the ALAS2 MD simulations (Figure 4C). However, incorporation of the HemAm data into this analysis resulted in noticeable loss of this correlation (Figure 4D). Furthermore, in comparison with the HemAm sets, the ALAS2 MD simulations had stronger correlation between the hydrogen bond number and the number of the β-strand-forming amino acids at the active site loop.

According to the linear regression analysis, there was a significant correlation between the mean hydrogen bond number and the mean number of β-strand forming residues in the analysis of the ALAS2 systems. Curiously, the homodimer asymmetry was also evident in this correlation analysis. In fact, two protomers showed different values of these parameters at the same temperature, although those systems (ALASdChA and ALASdChB) originated from the same MD simulation set at each temperature. There were much greater differences between A and B chains of the ALAS2 homodimer in terms of the number of β-strand-forming residues.

### 2.5. Anti-Correlation between the Active Site Loop and the C-Terminal Tail of Murine ALAS2

Clustering analysis revealed that the representative structures of top 3 clusters of ALASm at 300 K have a β-strand formed at the active site loop, although it is missing in clusters at 288 K or 310 K. However, ALASdChA and ALASdChB showed the presence of the β-strand at the active site loop only at 310 K and 288 K, respectively, although in the homodimer simulation at the same temperature, another protomer did not have the corresponding β-strand (Supplementary, Figure S2).

To further analyze the homodimer structural asymmetry focusing at the dynamics of active site loop and its relation to the structural dynamics of C-tail, we generated the cross-correlation heat maps based on the Pearson correlation coefficients obtained from the co-variance analysis of the C_α_ atom dynamics, thereby substantiating our original hypothesis. As a matter of fact, anti-correlation was observed for all of the MD simulations of ALASm conducted at the three different temperatures. This anti-correlation appeared to increase with an increase in temperature (Supplementary, Figure S3). Hence, the MD simulation for ALASm at 300 K showed the intermediate anti-correlation levels among all of the MD simulations generated for ALASm at three different temperatures. The ALAS2 dimer was characterized by the opposite behavior of its two protomers. At 288 K, neither protomer showed a strong anti-correlation between the dynamics of their active site loop and C-terminal tail. However, the MD simulations at both 300 K and 310 K yielded different trends. At 300 K, ALASdChA showed a strong anti-correlation, whereas the anti-correlation was noticeably weaker in ALASdChB.

**Figure 5:**
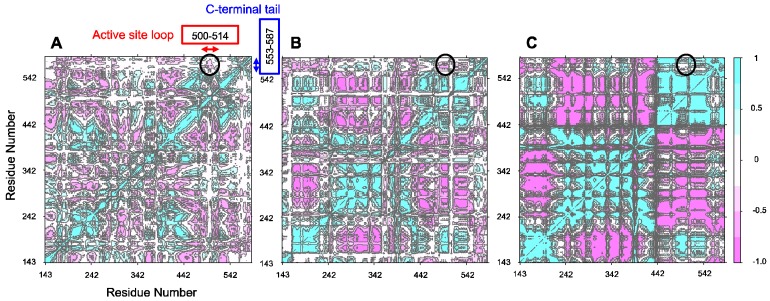

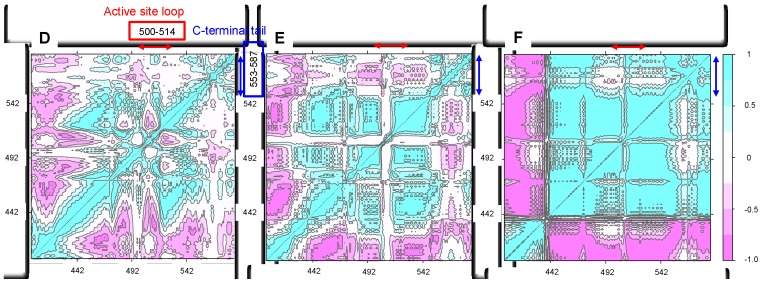
Correlation maps for the MD simulations of the wild-type C-terminal tail-containing ALAS2 systems at 300 K. (**A**) ALASm, (**B**) ALASdChA, (**C**) ALASdChB. 288 K, and 310 K correlation maps are provided in Supplementary, Figure S3. Black circles indicate the positioning of the anti-correlated data. (**D**–**F**). Zoomed in versions of (**A**–**C**), respectively.

**Figure 6:**
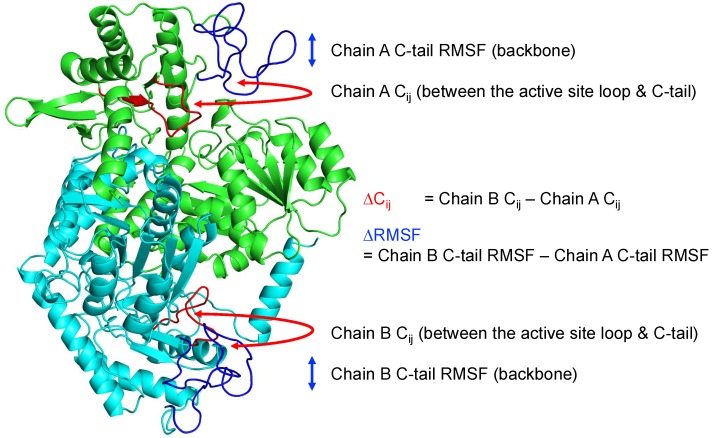
Representative structure of the ALAS2 homodimeric form observed in the molecular dynamics simulation. ∆C_ij_, and ∆RMSF measurement description is provided in a diagram. Red part of protein structure represents the active site loop, and blue part represents C-terminal tail (C-tail) in both protomers (Green: Chain A, Cyan: Chain B).

In contrast, at 310 K, ALASdChA showed a weak anti-correlation, but the anti-correlation was strong in ALASdChB. Curiously, both systems share the same general trend, where if one protomer shows a strong anti-correlation, the other protomer elicits a weak anti-correlation at the same temperature. This structural anti-correlation is illustrated in Figure 5, which shows the correlation maps for the MD simulations of the wild-type C-terminal tail-containing ALAS2 systems at 300 K. An illustrative example of such structural anti-correlation is given by Figure 6, representing the structure of ALAS2 homodimeric form and clearly showing that when an active site loop forms a β-strand in one protomer, it retains an irregular structure in another protomer.

From the backbone RMSF analysis, it was expected that MD simulations of ALASmWoT and HemAm would indicate that the N-terminal helix correlated with some other regions these proteins, which are devoid of the wild-type C-terminus (Supplementary, Figure S4). To check if this assumption was correct, we focused on the dynamic behavior of the N-terminal helix and on the dynamics of the active site loop. While a correlation was determined, the values were less prominent in the truncated proteins than in those with the wild-type C-terminus (or C-tail).

Therefore, to see in detail structural asymmetry in a homodimer based on the active site loop—C-terminal tail anti-correlation, and its relation to backbone RMSF—we applied linear regression between ∆C_ij_, and ∆RMSF as described in Section 4.2, focusing on the C-terminal tail (Figure 6). After the linear regression analysis, it was found that all of the homodimer structures in simulation have significant negative correlation between C-terminal ∆C_ij_, and ∆RMSF. The slopes of all analyses were negative, and each corresponding p-value was less than 0.05. Table 1 summarizes the results of the linear regression analysis. This analysis shows that if the C-tail fluctuation of one protomer is greater than in another protomer of a homodimer due to structural asymmetry, its correlation coefficient becomes more negative, thereby reflecting stronger anti-correlation.

One could ask the question as to why the anti-correlation of this pair of motifs is more important than other pairs, some of which are significantly more (anti)correlated than this subset of structural motifs. To answer this question, one should keep in mind that the main function of ALAS2 is to synthesize ALA, which is the first and rate-limiting step in heme biosynthesis. The dynamics of the active site loop is an important factor for controlling the rate of ALAS2-catalyzed reaction. In fact, ALAS2 mutant with the hepta-variant of the active site loop showed faster reaction than the wild-type protein, and in the hepta-variant molecular dynamic simulations, we observed that the β-strand is periodically formed in the active site loop. Since ALAS2 mutations with the modified C-tail cause XLPP associated with the “gain-of-function” and consequent overproduction of protoporphyrin IX and increase in Zn^2+^–protoporphyrin levels, we hypothesized that the dynamics of C-tail could affect β-strand formations at the active site loop. In other words, it is likely that the C-tail is important for the ALA synthesis being structurally associated with the rate-determining and β-strand-forming active site loop.

## 3. Discussion

Our previous MD simulation study on a functionally enhanced hepta-variant of mouse of ALAS2 and the wild-type enzyme revealed a dynamic structural transformation in the active site loop [47]. In particular, an emerging β-strand in the active site loop interacted with two preexisting β-strands forming an anti-parallel three-stranded β-sheet [47]. Although the focus of our previous study was on the structural changes undergone by the ALAS2 hepta-variant, we noticed that the loop-to-β-strand transition also occurred in the wild-type ALAS2 dimer, albeit less frequently than in the ALAS2 hepta-variant. Furthermore, preliminary visual inspections of the obtained MD-simulated structures revealed that when the β-strand formed in the ALAS2 active site loop, the C-terminal tail of the molecule became more dynamic. The current study was conceived based on these preliminary observations and aimed to attain a detailed analysis of the structural anti-correlation between the active site loop and the highly disordered C-terminal tail of ALAS2. As an IDPR, the ALAS2 C-terminal tail has a highly dynamic nature and a low folding energy. However, we identified proteins, like *M. thermoautotrophicum* phosphoribosyl-AMP cyclohydrolase HisI, with a C-terminus highly similar in sequence to that of ALAS2, but yet structured (Figure 1). Therefore, we raised the question of whether the dynamics of the ALAS2 C-terminal tail relate to those of the functionally and structurally important active site loop. Because of the enhanced enzymatic activity and substrate specificity of ALAS2 “hepta-variant” [21], we considered the loop-to-β-strand transition in the active site loop to be “a promoting factor” of the ALAS-catalyzed reaction [47]. Consequently, we hypothesized that the C-terminal tail folding dynamics affects the ALAS2 active site loop by contributing to the β-strand formation in this region. A structural and dynamic anti-correlation between the ALAS2 mobile active site loop and the highly disordered C-terminal tail would provide strong supporting evidence for this hypothesis.

To search for such potential anti-correlation, we performed MD simulations at three different temperatures (288 K, 300 K, and 310 K) of four different ALAS systems: HemA monomer (HemAm), ALAS2 monomer without the wild-type C-terminal tail (ALASmWoT), wild-type ALAS2 monomer (ALASm), and the wild-type ALAS2 homodimer. Although the wild-type ALAS2 dimer was used in MD simulation, the structural and dynamic characteristics of its protomers were analyzed separately as ALASdChA and ALASdChB sets. The backbone RMSD analysis revealed that the C-terminal tail is a highly dynamic entity (Figure 2), as was expected from its high predisposition for intrinsic disorder (Figure 1). For all systems analyzed in this study, the RMSF analysis revealed that the conserved region (residues R19-A409) is characterized by lower fluctuation levels than other protein regions (Figure 3). On the other hand, the C-terminal regions of ALASdChA, ALASdChB, and ALASm showed the greatest degree of fluctuation relative to the remaining protein parts. When the C-terminal tail was missing (as in HemAm and ALASmWoT), the resulting systems showed greater fluctuation of their N-terminal regions (which are also predicted to be disordered, see Figure 1). This means that at least one of the two disordered tails of ALAS is always in a dynamic state. We can also hypothesize that the loss of the ALAS C-terminal tail causes an increase in the fluctuation of the N-terminal tail as a compensatory mechanism. Further, in the C-tail-less ALAS systems, the flexibility of the N-terminal regions plays a role in maintaining the structural stability of functional conserved region, because of the dynamic nature of the flexible tail not much of energy is used to stabilize it, and the remaining folding energy could be distributed over the conserved region, thereby enhancing its structural stability.

Analysis of the number of hydrogen bonds and the number of β-strand-forming amino acids in the different ALAS2 systems showed that the active site loop of wild-type ALAS2 can form a β-strand form. Currently, there is no structural information related to the full-length mammalian ALAS protein. The only currently available ALAS structure was obtained for the bacterial protein, *R. capsulatus* HemA [3]. However, the bacterial protein structure did not show the β-strand formation in any structurally characterized forms, such as HemA alone (PDB ID: 2BWN), in a complex with succinyl-CoA (PDB ID: 2BWO) or in a complex with the glycine substrate (2BWP). One of the possible reasons for this observation is that *R. capsulatus* HemA does not need a loop-to-β-strand transition in its active site loop due its structural stability. In fact, the HemA active site loop shows a greater number of hydrogen bonds than that of ALAS2, although it has a relatively lower number of the β-strand forming residues. This might suggest that the active site loop of HemA has a sufficient number of hydrogen bonds and does not need to form an additional β-strand for optimal enzymatic activity. On the other hand, in comparison with HemA, the ALAS2 systems showed a lower number of hydrogen bonds but a greater number of the β-strand-forming residues. Therefore, we evaluated the correlation between the number of hydrogen bonds and the number of the β-strand-forming residues solely in the ALAS2 and HemA systems (Figure 4C,D). This analysis revealed that the correlation (with a more significant *p*-value) between the number of hydrogen bonds and the number of β-strand-forming residues in the ALAS2 systems is greater than in all HemA systems. This means that the formation of a β-strand in the ALAS2 active site loop is more strongly dependent on the number of hydrogen bonds than in the HemA active site loop.

In its mature form, bacterial ALAS exists as a tightly interlocked homodimer, the structure of which is stabilized by the contribution of all three domains (an N-terminal domain (residues 1–52), a central, catalytic domain (residues 53–296) contributing most of the dimer interface, and a C-terminal domain (residues 297–401)) of each protomer [3]. It was pointed out that some homodimers and higher-order homooligomers might have an asymmetric structure [50,51,52,53,54,55]. This homodimer asymmetry is thought to be involved in the “half-of-the sites” reactivity and self-assembly, and also defines some unique dynamic properties by generating multiple conformations of the dimer, variable self-assembly patterns, and adaptive protein recognition [52]. From the enzymology perspective, such homodimer asymmetry (or lack of strict structural equivalence between subunits) can be related to allosteric regulation, where intrinsic disorder may play a role in optimization of the allosteric coupling in proteins [56,57,58,59,60]. In fact, it was pointed out that the presence of intrinsic disorder in the domains or segments containing one or both of the coupled binding sites maximizes the site-to-site allosteric coupling [56]. In some cases, such allosteric control is determined by protein terminal regions, which are partially or completely disordered [58,60]. Recently, the role of homodimer asymmetry in allosteric pathways was assessed for a bacterial homodimeric enzyme, fluoroacetate dehalogenase (FAcD) [61]. Using X-ray crystallography, NMR, and MD simulation, it was shown that catalysis was associated with enhanced conformational exchange rate in the protomer and water release. This compensated for the entropic losses caused by the substrate binding and facilitated sampling of the transition state [61].

In line with these observations, our analysis revealed the presence of a noticeable ALAS2 homodimer asymmetry caused by structural and dynamic differences between the two protomers. This asymmetry was especially evident based on the analysis of the number of β-strand-forming residues. In fact, ALASdChA and ALASdChB showed opposite patterns of changes in their numbers of β-strand-forming residues (Figure 4). At 288 K, ALASdChA possessed a lower number of such residues in the active loop than ALASdChB, but at 300 K and 310 K, ALASdChA showed a greater number of β-strand-forming residues than the ALASdChB did. This means that when one protomer has a low number of β-strand-forming residues, another protomer contains a greater number of such residues. Since in our previous study we showed that the β-strand formation caused more efficient enzymatic reaction, the found asymmetry can be related to the aforementioned “half-of-the-sites reactivity” model of the homodimer (or, more generally, homooligomer) functioning [47,52,62]. Here, the model describes activity of proteins composed of identical subunits with *n* potential active sites that react with their substrates in such a way that only half of sites are occupied when the enzyme is saturated with the ligand [62]. It was proposed that the potential explanation for this phenomenon is in the presence of the extreme negative cooperativity, where the conformational changes induced by a ligand in one protomer are transmitted to the neighboring protomer to prevent its interaction with the ligand [62].

Our MD simulation data provide an interesting dynamics-based foundation for this phenomenon by showing that the dynamic and related structural asymmetry is present already in the absence of ligands. Furthermore, the asymmetry in the dynamic behavior of protomers is complicated by the presence of a clear anti-correlation in the intramolecular dynamics of their different parts, the active site loops and the C-terminal tails. In other words, protomers in our study have shown structural and dynamic dissimilarities at two different levels, between each other and between different parts within each protomer. The presence of such “double impact” may lead to the presence of unique interactions between the two protomers that produce the overall effect seen in the actual dimer—even though their structures are very similar, they act differently. In other words, the presence of structural variations between the homodimers coupled with the anti-correlation in their intraprotomer dynamics may depend on some specific dimeric interactions.

This study showed the potential involvement of intrinsically disordered C-terminal tail in the structure, molecular dynamics, and function of ALAS2, and suggests the functional contribution of IDPRs in the activity of various enzymes. Furthermore, the usefulness of IDPRs as potential drug targets should also be considered. In fact, a small molecule, 10058-F4, which is a c-Myc-Max inhibitor, serves as an illustrative example of the drug lead causing the disorder-to-order transition in a target protein [63,64,65]. This molecule was discovered through the yeast two-hybrid experiments. It was subsequently shown that binding of 10058-F4 to a specific site of c-Myc causes misfolding of this protein via the induced disorder-to-order transition [63,64]. Therefore, the action mechanism of 10058-F4 is based on the drug-induced misfolding of intrinsically disordered c-Myc leading to the direct inhibition of c-Myc-Max interaction [63,64]. It is of interest to consider a possibility of finding a similar molecule affecting the structure of the ALAS2 C-terminal tail, even though the molecular functions and structures of this ALAS2 region are different from those of c-Myc. From the results of this study describing the anti-correlation between the structural and dynamic behavior of the ALAS2 active site loop and the C-terminal tail, it is tempting to hypothesize that it would be possible to discover a drug molecule targeting the C-terminal region of ALAS2 and forcing this region to fold, thereby modulating flexibility of the ALAS2 active site loop. Once this loop is more flexible, the reaction rate of ALAS2 would decrease. Therefore, it is expected that it would be possible to control ALAS2 enzymatic efficiency through the discovery of a ligand causing induced folding of the C-terminal tail of this protein. Since XLPP is caused by the enhanced reaction rate of ALAS2 [11], discovery of such a ligand would be a good starting point for the development of a new drug against XLPP.

The roles of intrinsic disorder in C- and N-terminal tails in formation of protein homo-oligomers were also highlighted in previous aggregation and amyloidogenesis studies. For example, analysis of the β2-microglobulin aggregation revealed that the enhanced dynamics of both tails caused more efficient aggregation of two protomers than aggregation in this protein form where the only C-terminal tail was flexible and even aggregation of previously discovered amyloidogenic mutants [66,67]. In the analysis of the dimerization of amyloidogenic variants of the α-spectrin SH3 domain (Spc-SH3), it was also discovered that the formation of partially folded intermediate where the N-terminal β1-strand is unstructured, and that a large part of the hydrophobic core is solvent exposed, is crucial for protein aggregation [68]. It was also pointed out that this unfolded strand β1 was a major driver of the Spc-SH3 dimerization process [68]. However, neither study analyzed the correlation between the dynamic behavior of disordered tail(s) and the ordered parts for the corresponding proteins.

Finally, one should keep in mind that higher levels of intrinsic disorder are frequently found in the tails of various proteins [69]. Therefore, it is reasonable to hypothesize that the correlation or anti-correlation in the dynamics of disordered tails with the dynamic behavior of other parts of proteins can serve as a unique control of the overall protein dynamics and functionality.

During the preparation of this manuscript for submission, an article was published describing the crystal structure of *Saccharomyces cerevisiae* ALAS from [70]. The overall fold of this protein was similar to that of *R. capsulatus* HemA, with both of these two structures being characterized by a backbone RMSD of ~1.1 Å for common Cα atoms [70]. The homodimeric structure of *S. cerevisiae* ALAS also illustrates the functional asymmetry of the enzyme, since the covalently bound PLP cofactor was only found in one of the two ALAS protomers [70]. However, one should keep in mind that the observed asymmetry in substrate binding could be a structural artifact, since sometimes PLP is lost during the protein purification. The authors also indicated that PLP binding promoted reordering of the active site and structural change of some other protein regions [70]. Finally, it was pointed out that the C-terminal tail is engaged in wrapping around the dimer and even can contact active-site-proximal residues [70].

## 4. Experimental Section

### 4.1. Sequence Analysis and Intrinsic Disorder Prediction

Amino acid sequence alignments between *R. capsulatus* ALAS or HemA (UniProt ID: P18079) and the mature form of the *Mus musculus* ALAS2 (UniProt ID: P08680, residues D79-A587) were performed using Clustal Omega 1.2.2 algorithm [71,72]. Searches of regions of similarity between protein sequences and calculations of the degree of similarity were accomplished with BLAST [72,73]. The intrinsic disorder predispositions and the average disorder scores for the different proteins (HisI and ALAS2) were evaluated using PONDR^®^ FIT, PONDR^®^ VLXT, and PONDR^®^ VSL2 disorder predictors [39,74,75,76,77].

### 4.2. Molecular Dynamics (MD) Simulation Analysis

The I-TASSER web server [78] was used to conduct homology modeling for ALAS2 F143 – A587 using the crystal structure of *R. capsulatus* ALAS (HemA, PDB ID: 2BWN, chain D [3]) as a template. To this end, the HemA monomer structure was modeled after the chain D of the PDB entry 2BWN, and the hydrogen atoms were added using the PDB2PQR web algorithm [79]. The LLP residue (*N*′-pyridoxyl-lysine-5′-monophosphate) structure was obtained from the template PDB file using PyMol 1.3 to prepare the ALAS2 structure. The LLP residue parameters were obtained using CGenFF [80]. The following structures were generated for the MD simulation analysis: HemA monomer (HemAm), ALAS2 monomer without the C-terminal tail (ALASmWoT), mature wild-type ALAS2 monomer (ALASm), and the wild-type ALAS2 homodimer containing two polypeptide chains (ALASdChA, ALASdChB).

GROMACS 5.0.4 double precision version, installed with FFTW 3.3.4 library [80,81], and CHARMM 36 force field developed by MacKerell Lab (http://mackerell.umaryland.edu/ charmm_ff.shtml, accessed on 10 July 2015) [82] were used in the MD simulations. A truncated octahedron box with a periodic boundary condition was constructed with a buffering region thickness of 1.2 nm. For explicit water models in the MD simulation systems, 55.6 mol/L TIP3P water molecules were constructed. Then, ~150 mM K^+^ and Cl^-^ ions were added to the systems [83]. The number of ions was adjusted to neutralize the system. Energy minimization using the steepest descent method was performed until the maximum force was less than 1000 kJ mol^−1^ nm^−1^.

For thermalization, canonical ensemble MD simulations (NVT, where the number of particles (N), volume (V), and temperature (T) are conserved) of 5 ns serial heating (Δt = 2 fs for each step and 500,000 steps for each 1 ns heating at certain temperature: 100 K, 150 K, 200 K, 250 K, and the final temperature) were conducted in this study. We set three different end temperatures, 288 K, 300 K, and 310 K. Thus, three replicas of each system were prepared. The temperature of 288 K was selected because we used this temperature in our previous experimental and computational studies [21,47]. A position restraint constant of 1000 kJ mol^−1^ nm^−2^ was applied to all heavy atoms, and V-rescale was used to control the temperature [84]. The lengths of bonds involving any heavy atom were constrained using the LINCS method [85]. For long-range electrostatics calculations, the Particle Mesh Ewald method (PME) was used [86,87]. The Verlet cut-off scheme was used to update the “neighbor list” every 10fs [88]. The electrostatic and the van der Waals cutoffs were set to be 1.2 nm.

For equilibration, isothermal-isobaric ensemble MD simulations (NPT, where the number of particles (N), pressure (P), and temperature (T) are kept constant) of 5 ns equilibration (Δt = 2 fs × 500,000 steps for each 1ns equilibration with certain position restraint constant: 1000 kJ mol^−1^ nm^−2^, 550 kJ mol^−1^ nm^−2^, 100 kJ mol^−1^ nm^−2^, 55 kJ mol^−1^ nm^−2^, and 10 kJ mol^−1^ nm^−2^) were employed. Parrinello-Rahman barostat was applied to maintain the pressure of the system at 1 bar [89,90]. Other conditions were the same as those in the heating step.

During the production runs, the NPT ensemble MD simulations of 100 ns (Δt = 2 fs for each step) were continued without position restraints. During each production step, 1001 frames were collected with the 100 ps intervals for each system, and the corresponding trajectories for HemAm, ALASmWoT, ALASm, ALASdChA, and ALASdChB were stored for further analysis. The ALAS2 dimer MD simulation trajectory was performed separately for chains ALASdChA and ALASdChB.

Protein backbone root-mean squared deviations (RMSD) and root-mean squared fluctuations (RMSF) in the MD simulation trajectory sets were calculated using GROMACS 5.0.4 ‘rmsdist’, and ‘rmsf’ functions. For ALASdChA, ALASdChB, and ALASm, the backbone RMSD calculations were conducted for three ALAS2regions: the N-terminal helix (residues F143-H160), conserved region (residues T161-V552), and C-terminal tail (residues A553-A587). However, the backbone (N-C_α_-C) RMSD calculations for the HemAm, and ALASmWoT chains, which lack the C-terminal tail, were conducted for two regions: the N-terminal helix (residues F143-H160 and M1-G18 in ALASmWoT and HemAm, respectively) and the conserved region (residues R19-A409 and T161-V552, in HemA and ALASmWoT, respectively). These regions were determined based on their structural homology with the HemA X-ray crystal structure [3]. RMSD distributions were plotted using the ‘generateFES.py’ algorithm provided by the Strodel lab (http://www.strodel.info/index_files/lecture/generateFES.py), and the SigmaPlot 11. Backbone (N-C_α_-C) RMSF calculations were plotted using R 3.2.2 [91]. Plots of backbone RMSD distribution for HemAm, ALASmWoT, ALASm, ALASdChA, and ALASdChB MD simulations conducted at different temperatures are shown in Supplementary, Figure S5, whereas Supplementary, Figure S6 represents time courses of total energy of system in HemAm, ALASmWoT, ALASm, and ALASd MD simulations conducted at different temperatures.

We applied GROMACS 5.0.4 “hbond” to measure the number of hydrogen bonds between the structurally conserved β13 strand (residues R372–F375 and L515–L518 in HemA and ALAS2, respectively) and the loop region (residues Y357–E371 and Y500–E514 in HemA and ALAS2, respectively) due to the presence of specific structural changes described in a previous study [47]. We also applied the “do_dssp” function to measure the number of β-strand-forming amino acids during the “hbond” measurement analysis. The ‘cluster’ function (linkage method) was applied to show the most probable structural characteristics of each system. Reference for the clustering was the energy minimized backbone structure of conserved regions (residues 19–397 and 161–540 in HemA and ALAS2, respectively). For cross-correlation matrix generation, each MD simulation set backbone trajectory was fit to its conserved region (residues T161–V552 for ALAS2, and residues R19–A409 for HemA) of the corresponding energy minimized structure. The R package Bio3D 2.2-0 “dccm” was used to generate the displacement cross-correlation matrix among the C_α_ atoms from the fitted backbone trajectory [91,92]. Bio3D “dccm” obtains co-variance matrices from given fitted coordinates internally, and it calculates Pearson correlation coefficients from co-variance matrices, invoking “cov2dccm”. Calculated cross-correlation coefficients (C_ij_) were plotted using “plot” of R (C_ij_ = 1, strong correlation between C_α_ atoms; C_ij_ = 0, no correlation between C_α_ atoms; C_ij_ = −1, strong anti-correlation between C_α_ atoms, where indices “I” and “j” indicate two nodes of the correlation matrices.

In case of the analysis of two protomers of homodimer trajectories (ALASdChA, ALASdChB), we measured residue level ∆Cij, and ∆RMSF to apply linear regression. ∆Cij was obtained from the active site loop (residues 500–514, mean values of 15 residues were taken), and C-tail (residues 553–587) Cij values (∆Cij = ALASdChB Cij − ALASdChA Cij). ∆RMSF was obtained from the C-tail (residues 553–587) RMSF values ((∆RMSF = ALASdChB RMSF − ALASdChA RMSF). Then, the values of two corresponding columns were analyzed using “lm” function in R.

## 5. Conclusions

To analyze the possibility of the existence of a structural and dynamic anti-correlation between the mobile active site loop and the highly disordered C-terminal tail of ALAS, a comprehensive MD simulation analysis was conducted at three different temperatures (288 K, 300 K, and 310 K) for several ALAS-related systems: HemA monomer (HemAm), ALAS2 monomer with a truncated C-terminal tail (ALASmWoT), mature wild-type ALAS2 monomer (ALASm), and the wild-type ALAS2 homodimer with its two protomers, ALASdChA and ALASdChB. This analysis revealed that the C-terminal tail, predicted to be a highly disordered region, was indeed the most mobile region of the ALASdChA, ALASdChB, and ALASm systems. A high level of conformational fluctuation was detected in the N-termini of HemAm and ALASmWoT, which have shorter or truncated C-termini when compared to ALAS2, respectively. The N-termini of these proteins had been predicted to be disordered, and thus our findings indicate that at least one of the two disordered termini or tails of ALAS is always highly conformationally flexible. The identification of a clear anti-correlation between the dynamics of the active site loop and those of C-terminal tail supported our hypothesis that the intrinsically disordered C-terminal tail plays an important role in the regulation of the dynamic behavior of the ALAS2 active site loop and thereby in the regulation of the function of ALAS2. We also showed that the wild-type ALAS2 homodimer is predominantly asymmetric, with its ALASdChA and ALASdChB protomers possessing different structural and dynamic properties. Although such an asymmetry has been reported for ligand bound-forms of several homo-oligomers and assumed to play a role in their “half-of-the-sites activity”, our MD simulation results lead us to suggest that the dynamic and structural asymmetry is present in such homo-oligomers even in the absence of ligands. In sum, our data demonstrate that the ALAS2 homodimer is characterized by structural and dynamic asymmetry of its protomers, with a distinct anti-correlation between the intramolecular dynamics of their active site loops and those of their C-terminal tails.

## Figures and Tables

**Table 1:** Linear regression coefficients (y=α+β x, where y is ∆C_ij_, and x is ∆RMSF).

Homodimer System	Intercept (α)	Slope (β)	*p*-Value
288 K	0.17	−0.40	0.04
300 K	0.42	−0.50	0.04
310 K	−0.04	−1.10	0.01

## Supplementary Materials

Supplementary materials can be found at http://www.mdpi.com/1422-0067/19/7/1899/s1.
