# Supplementary material for: Anti-Correlation between the Dynamics of the Active Site Loop and C-Terminal Tail in Relation to the Homodimer Asymmetry of the Mouse Erythroid 5-Aminolevulinate Synthase
Source: Int J Mol Sci. 2018 Jun 28;19(7):1899. doi: 10.3390/ijms19071899 (PMC6073955; doi:10.3390/ijms19071899)
Supplement: Supplementary file 1 [file ijms-19-01899-s001.pdf]

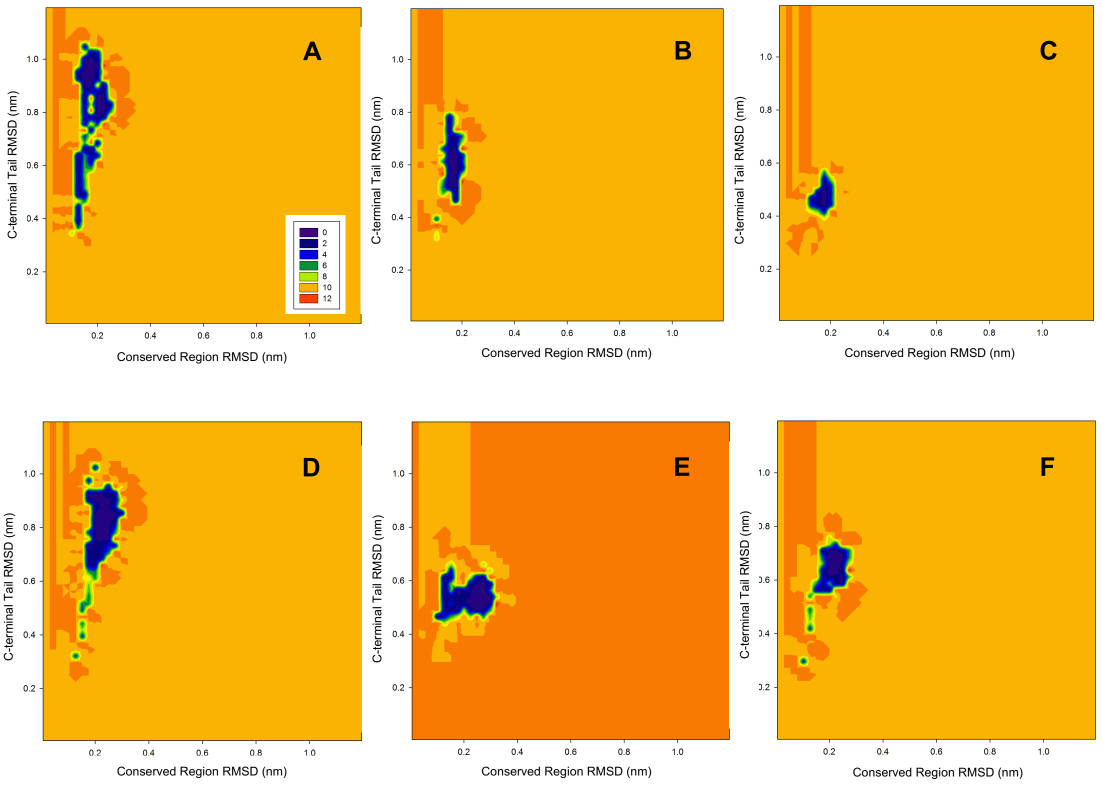

Supplementary Figure 1. RMSD distribution of the MD simulations for the different ALAS2 systems. A. ALASm at 288K. B. ALASdChA at 288K. C. ALASdChB at 288K. D. ALASm at 310K. E. ALASdChA at 310K. F. ALASdChB at 310K.

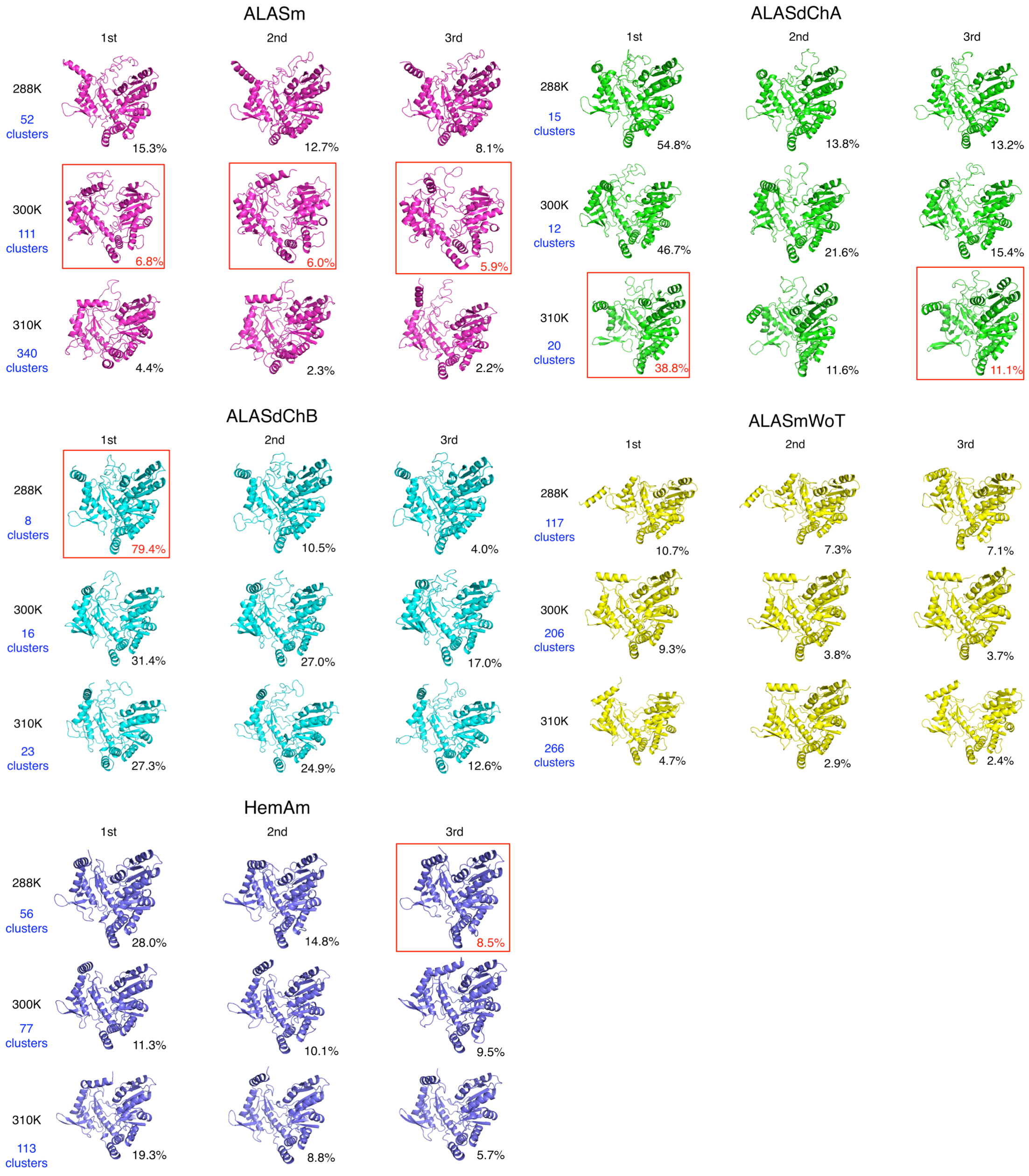

Supplementary Figure 2. Top 3 best clustering representative structures for the MD simulation trajectories. Red box indicates the representative structure of each cluster, where the active site loop shows β-strand structure. Blue letters indicate the total number of clusters. Percentage coverages from trajectory of each cluster is also described at the bottom of each cluster's representative structure.

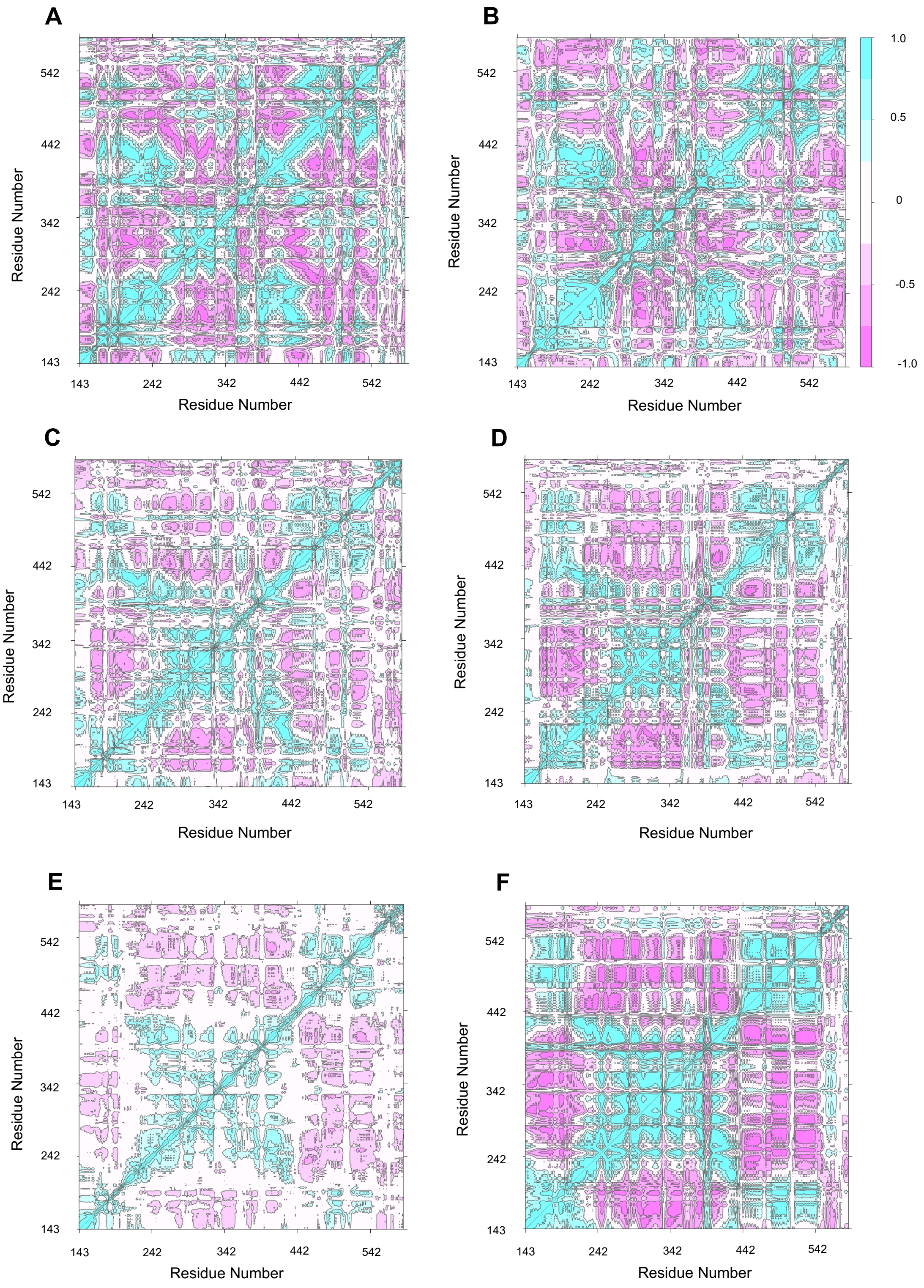

Supplementary Figure 3. Correlation map for the various wild-type C-terminal tail-containing ALAS2 sets. A. ALASm at 288K. B. ALASm at 310K. C. ALASdChA at 288K. D. ALASdChA at 310K. E. ALASdChB at 288K. F. ALASdChB at 310K. The color code scale indicates cross-correlation coefficients (Cij).

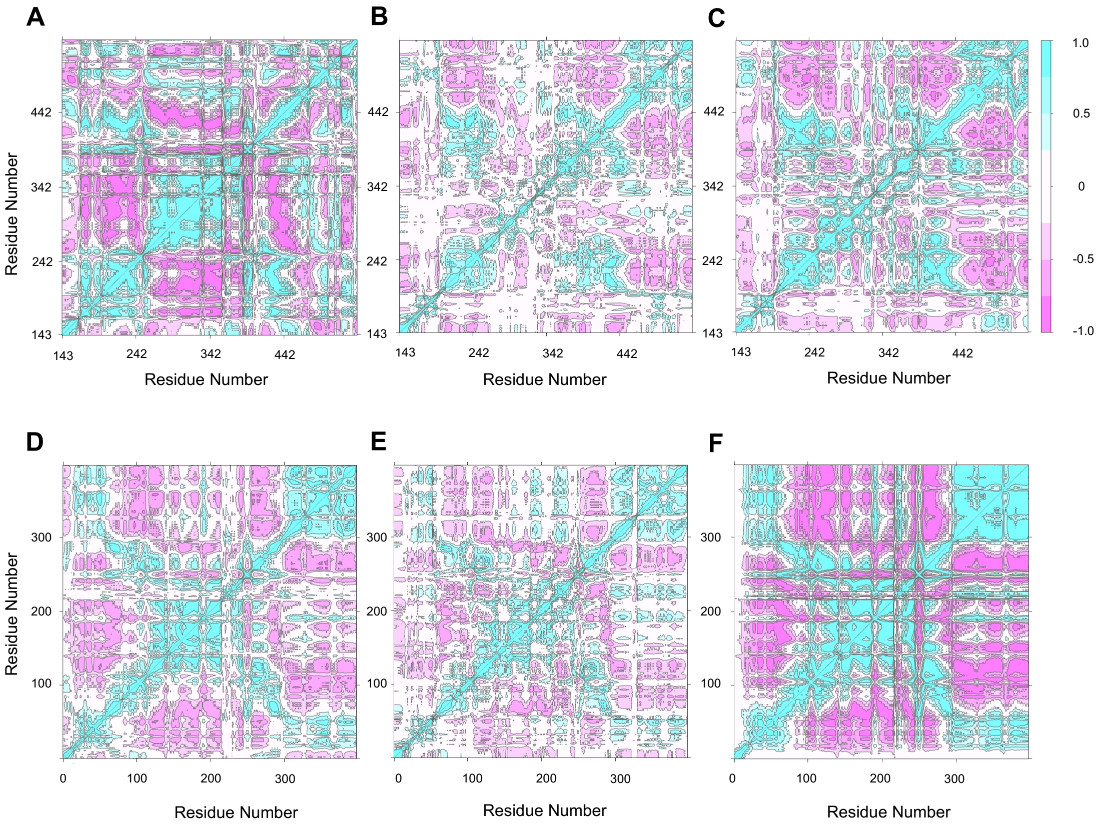

Supplementary Figure 4. Correlation map for ALASmWoT, and HemAm, which have a truncated C-teriminus and thus do not have the wild-type C-terminal tail. A. ALASmWoT at 288K. B. ALASmWoT at 300K. C. ALASmWoT at 310K. D. HemA at 288K. E. HemAm at 300K. F. HemAm at 310K. The color code scale indicates cross-correlation coefficients (Cij).

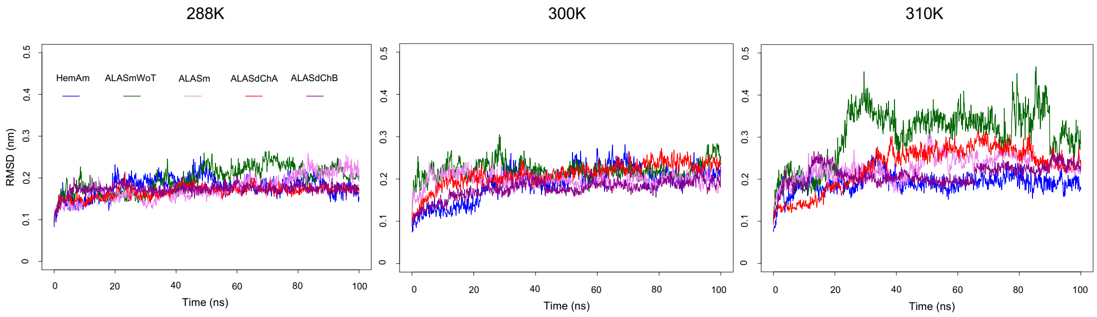

Supplementary Figure 5. Conserved region backbone RMSD in various MD simulations performed at different temperatures.

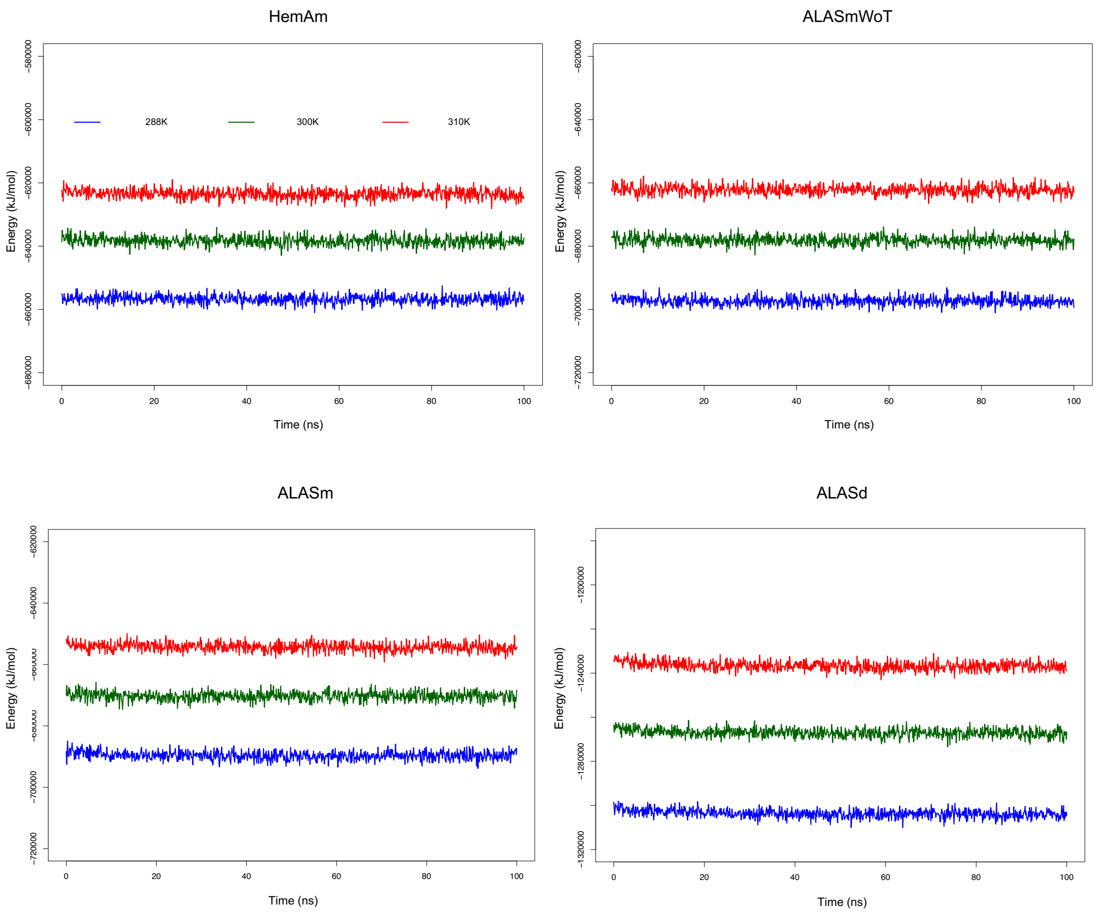

Supplementary Figure 6. Total energy of systems in different MD simulations.
